# 295. SARS-CoV-2 Associated Multisystem Inflammatory Syndrome in Adults (MIS-A): a Multicenter Case Series.

**DOI:** 10.1093/ofid/ofac492.373

**Published:** 2022-12-15

**Authors:** Ricardo Trigueros, Travis Denmeade, Danielle Pannebaker, Ryan C Maves

**Affiliations:** Wake forest Baptist Medical Center, Madison; Wakeforest Baptist Medical center, winston salem, North Carolina; San Diego Naval Medical Center, san diego, California; Wake Forest University School of Medicine, Winston-Salem, North Carolina

## Abstract

**Background:**

MIS-A frequently presents with symptoms including fevers, cardiovascular, gastrointestinal mucocutaneous, and neurologic involvement in both children and adults. Predominantly occurs in previously healthy children and young adults, disproportionately affecting individuals of African,Latino and Asian descent. Represents one of the most significant and yet under-recognized clinical consequences of SARS-CoV-2 infection.

**Methods:**

A retrospective review was performed of the identified MIS-A cases admitted to two institutions between January 2020-December 2021. Included cases required evidence of present or previous SARS-CoV-2 infection by either PCR or antigen testing; and meeting the CDC definition for MIS-A. After case identification, we collected clinical and laboratory data to analyze demographics, clinical symptoms, laboratory result, imaging, length of hospitalization and therapeutic interventions.

**Results:**

Ten cases were identified in the established period of time. The average interval to hospital admission was 31days. Clinical symptoms included fever, malaise, gastrointestinal complaints, dyspnea, diffuse rash. Males outnumbered females in a ratio of 7:3,with African American and Latino patients comprising 80% of the cases. All patients had elevated inflammatory markers and the most commonly manifesting organ dysfunction was cardiovascular system.

**Figure:**
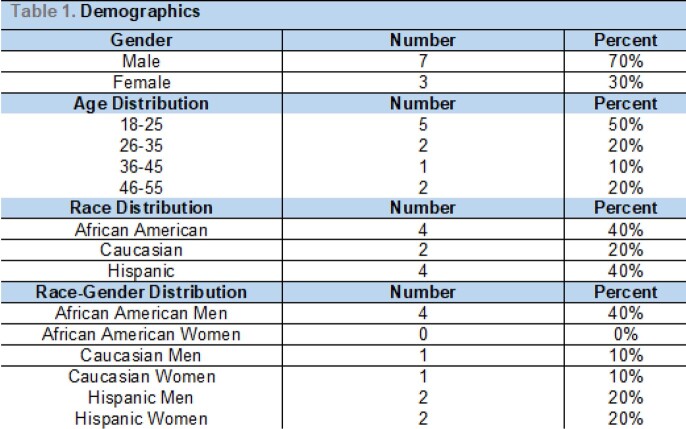

**Figure:**
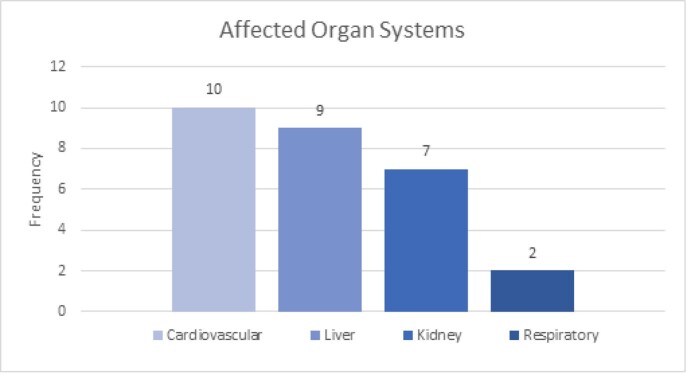

**Figure:**
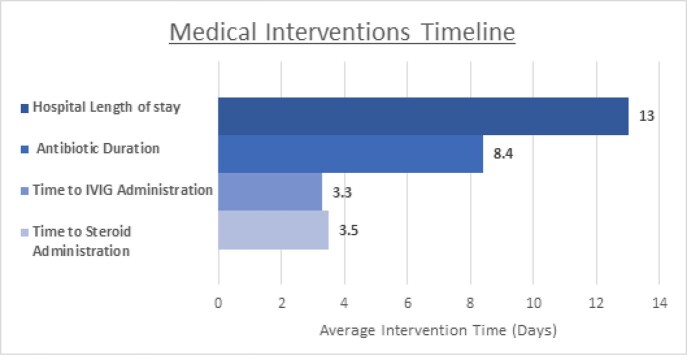

**Conclusion:**

This case series helps reinforce the characterization of an as yet incompletely understood and challenging clinical syndrome, providing insight to guide diagnosis and treatment of MIS-A. A greater understanding of MIS-A may facilitate earlier syndromic recognition in order to improve outcomes, reduce unnecessary antimicrobial use, and reduce costs.

**Figure:**
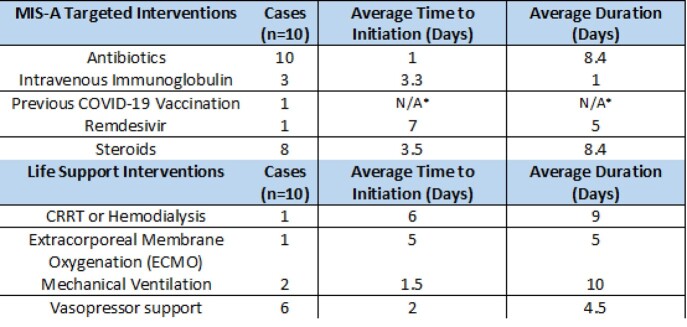

**Disclosures:**

**Ryan C. Maves, MD**, AiCuris: Grant/Research Support|Sound Pharmaceuticals: Grant/Research Support|Trauma Insights, LLC: Advisor/Consultant.

